# Associations between genetically predicted sex and growth hormones and facial aging in the UK Biobank: a two−sample Mendelian randomization study

**DOI:** 10.3389/fendo.2023.1239502

**Published:** 2023-10-17

**Authors:** Mingjian Zhao, Huiyun Lv, Yunshu Zhang, Hongliang Zhao, Hongzhi Qin

**Affiliations:** ^1^ Graduate School, Dalian Medical University, Dalian, Liaoning, China; ^2^ Department of Oncology, The Second Hospital of Dalian Medical University, Dalian, Liaoning, China; ^3^ Clinical Laboratory of Integrative Medicine, The First Affiliated Hospital of Dalian Medical University, Dalian, Liaoning, China; ^4^ Department of Burns and Plastic Surgery, Miyun Hospital, Capital Medical University, Beijing, China

**Keywords:** hormones, facial aging, mendelian randomization analysis, SMR analysis, drug target genes

## Abstract

**Background:**

Aging is an inescapable process, but it can be slowed down, particularly facial aging. Sex and growth hormones have been shown to play an important role in the process of facial aging. We investigated this association further, using a two-sample Mendelian randomization study.

**Methods:**

We analyzed genome-wide association study (GWAS) data from the UK Biobank database comprising facial aging data from 432,999 samples, using two-sample Mendelian randomization. In addition, single-nucleotide polymorphism (SNP) data on sex hormone-binding globulin (SHBG) and sex steroid hormones were obtained from a GWAS in the UK Biobank [SHBG, *N* = 189,473; total testosterone (TT), *N* = 230,454; bioavailable testosterone (BT), *N* = 188,507; and estradiol (E2), *N* = 2,607)]. The inverse-variance weighted (IVW) method was the major algorithm used in this study, and random-effects models were used in cases of heterogeneity. To avoid errors caused by a single algorithm, we selected MR-Egger, weighted median, and weighted mode as supplementary algorithms. Horizontal pleiotropy was detected based on the intercept in the MR-Egger regression. The leave-one-out method was used for sensitivity analysis.

**Results:**

SHBG plays a promoting role, whereas sex steroid hormones (TT, BT, and E2) play an inhibitory role in facial aging. Growth hormone (GH) and insulin-like growth factor-1 (IGF-1) levels had no significant effect on facial aging, which is inconsistent with previous findings *in vitro*.

**Conclusion:**

Regulating the levels of SHBG, BT, TT, and E2 may be an important means to delay facial aging.

## 1 Introduction

Facial aging is a multifactorial process governed by intrinsic and extrinsic factors that involves all tissues of the face, including the skin, muscles, fat, ligaments, and bone (1, 2). Accordingly, exploring the mechanisms involved in facial aging, particularly facial skin aging, has been an area of interest, not only for aesthetic purposes but also because they may provide mechanistic insights into diseases with similar mechanisms (3). In the current society, the greatest efforts are made to camouflage signs of facial aging (4–7). While our understanding of aging has evolved over the years, a comprehensive understanding of all contributing factors is still lacking.

Different molecular mechanisms have been suggested to explain facial aging. In recent years, the relationship between sex hormone-binding globulin (SHBG) and sex steroid hormones and facial aging has received extensive attention. The skin is the largest hormonally sensitive organ in the human body (8). Studies have shown that keratinocytes, Langerhans cells, melanocytes, sebaceous glands, and fibroblasts are affected by hormones (9). A pilot observational study of subjects who were 5 years into menopause revealed that long-term hormone therapy users had less severe wrinkling (10). During menopause, collagen loss accelerates due to the decrease in estrogen levels, with an average decline of 2.1% in skin collagen per postmenopausal year (11). In women on hormone therapy, collagen levels increase, and estradiol (E2) may play a role in collagen synthesis and the maintenance of hyaluronic acid levels (12).

Mendelian randomization (MR) is an epidemiological method that employs genetic variants as instrumental variables to proxy an exposure variable of interest and study the effect of the exposure on a certain outcome (3, 13). In this study, we aimed to examine the potential causal associations between SHBG, total testosterone (TT), bioavailable testosterone (BT), E2, growth hormone (GH), and insulin-like growth factor-1 (IGF-1) and facial aging using MR analysis (14) of data collected from the UK Biobank. MR is an ideal tool for investigating aging-related processes because genetic variables can affect lifetime when exposed to external environmental factors. We employed several MR methods to estimate the causal effects of sex hormones on the risk of facial aging and we used summary data-based Mendelian randomization (SMR) analysis to determine whether hormone-related drug target genes cause facial aging.

## 2 Materials and methods

### 2.1 Two-sample MR analysis

To investigate the effect of sex hormone levels on the risk of facial aging, we applied a two-sample MR approach. Single-nucleotide polymorphisms (SNPs) associated with facial aging in *SHBG*, *BT*, *TT*, *E2*, *GH*, and *IGF1* were obtained from a public genome-wide association study (GWAS) database. To ensure reliable results, the MR analysis satisfied the following three hypotheses: (1) the SNPs finally included must be closely related to SHBG, sex steroid hormones, GH, and IGF-1; (2) the SNPs and confounding factors included (related hormones and facial aging) are independent of each other; and (3) horizontal pleiotropy is not present, i.e., the SNPs affect facial aging only through the above hormones. The present study only used GWAS datasets from publicly available databases, and the authors who uploaded the data provided ethical approval in the original articles. Therefore, ethical approval was not required.

### 2.2 Sex hormone-related GWAS data collection

TT, BT, E2, and SHBG GWAS data were collected from the MRC Integrative Epidemiology Unit GWAS database (https://gwas.mrcieu.ac.uk/), using the R package “TwoSampleMR” (version 0.5.6). The SHBG, TT, and BT data were generated by Ruth et al. (15) and comprised 370,125, 194,453, and 178,782 samples, and 161,317,172, 16,131,612, and 16,131,701 SNPs under study accessions ebi-a-GCST90012111, ebi-a-GCST90012113, and ebi-a-GCST90012103. The E2 data were generated by Schmitz et al. (16) and comprised 163,985 samples and 748,8193 SNPs under study accession ebi-a-GCST90020092. The IGF1 and GH data were generated by Prins et al. and Folkersen et al. (17, 18) and comprised 9,732 and 21,758 samples under study accessions ebi-a-GCST005071 and ebi-a-GCST90012032.

### 2.3 Facial aging GWAS data collection

Data on facial aging were obtained from a publicly available GWAS database and included phenotypes and biological samples from 432,999 participants in Great Britain. The facial aging data in the UK Biobank were obtained via a questionnaire and can be accessed on the Integrative Epidemiology Unit GWAS database web site via accession ukb-b-2148.

### 2.4 SMR analysis of sex hormone drug-related target genes

Sex hormone-related drugs (testosterone undecanoate, testosterone, methyltestosterone, progesterone, estradiol benzoate, estrone sulfate, and estradiol acetate) and target genes of hormone-related drug action were obtained from the drug bank (https://go.drugbank.com/). Expression quantitative trait loci (eQTL) summary data were obtained from the eQTLGen Consortium (https://www.eqtlgen.org/) and comprised 31,684 individuals and 10,317 trait-associated SNPs. SMR analysis was performed using SMR-1.3.1 for Linux (19) with a screening threshold of 5E10^–8^ for SNPs and using default software parameters, with Bonferroni correction for multiple *p*-values.

### 2.5 Data analysis

All statistical analyses were performed using R software (version 4.2.0). The R package “TwoSampleMR” was used for MR analysis of the causal relationships between hormones and facial aging. SNPs were screened from aggregate data of the above-mentioned hormones, with the threshold set to *p* < 5E10^–8^. However, because of the low prevalence of SNPs in *E2*, *GH*, and *IGF1*, the screening threshold was relaxed to *p* < 5E10^–6^. Quality control and linkage disequilibrium (LD) analysis were performed to satisfy the MR hypothesis (*r* ^2^ < 0.001, aggregation distance = 10,000 kb) and remove palindromic SNPs. Because estimates tend to be biased toward null when weak SNPs are used in two-sample MR analysis, *F* > 10 was used to remove weak SNPs. The R package “MR-PRESSO” was used to remove outliers to ensure reliable results. *p* < 0.05 was considered statistically significant for evidence of potential causal effects. For multiple SNPs, the random-effects inverse-variance weighted (IVW) method was used as the primary estimator in MR analysis (20). Fixed/random-effects models were selected for the IVW test according to the existence of heterogeneity. In general, the IVW method assumes that all SNPs are valid instrumental variables. It is the most recognized method, and has high statistical power. The weighted median (21), MR-Egger regression (22), and simple and weighted mode methods were used for complementary analysis. Odds ratios (ORs) and 95% confidence intervals (CIs) were used to indicate the strength of the effect.

### 2.6 Sensitivity analysis

The R package “MR-PRESSO” was used to perform sensitivity analysis and remove outliers. Heterogeneity was tested using the Cochran *Q* test, and *Q* < 0.05 is considered absence of heterogeneity. Horizontal pleiotropy was assessed based on the MR-Egger intercept, and *p* < 0.05 was considered to indicate horizontal pleiotropy. In addition, leave-one-out tests were used for sensitivity analysis.

## 3 Results

**Figure 1:**
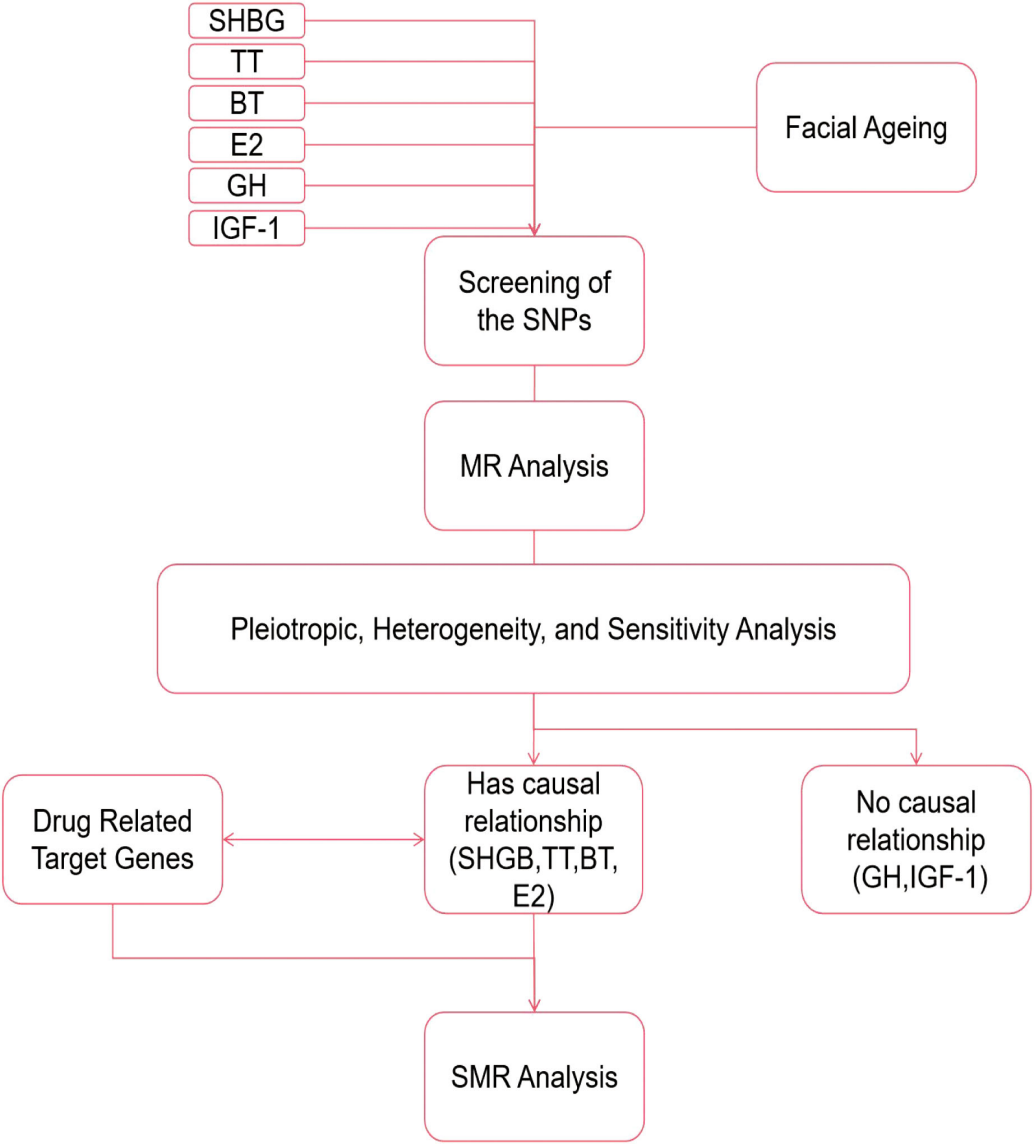
Schematic diagram of the analysis process.

**Figure 2:**
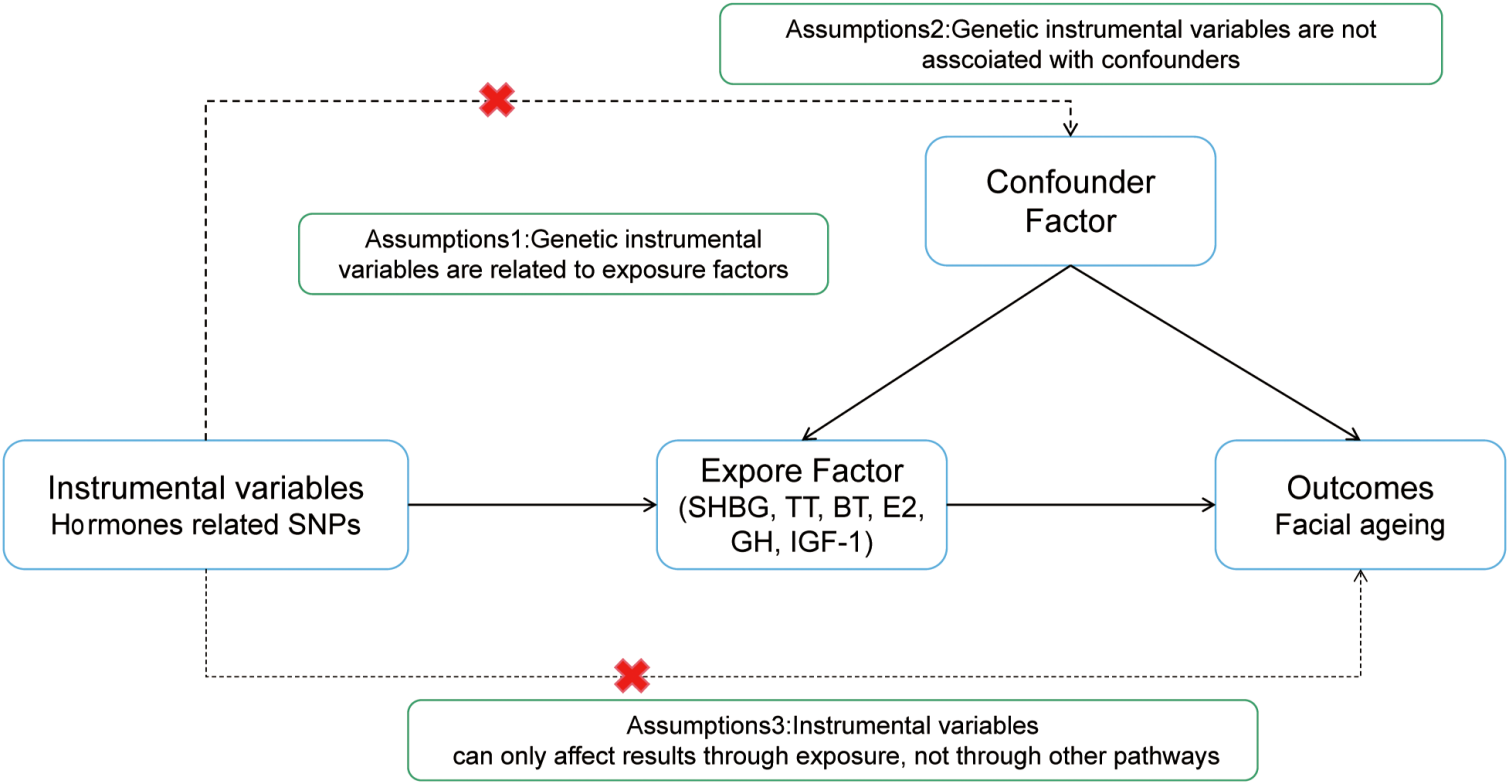
MR basic requirements framework. Two-sample MR studies need to satisfy three assumptions.

The simple flowchart of the research and the three assumptions of MR are shown in **Figures 1**, **2**.

### 3.1 Two-sample MR analysis of SHBG and facial aging risk

**Figure 3:**
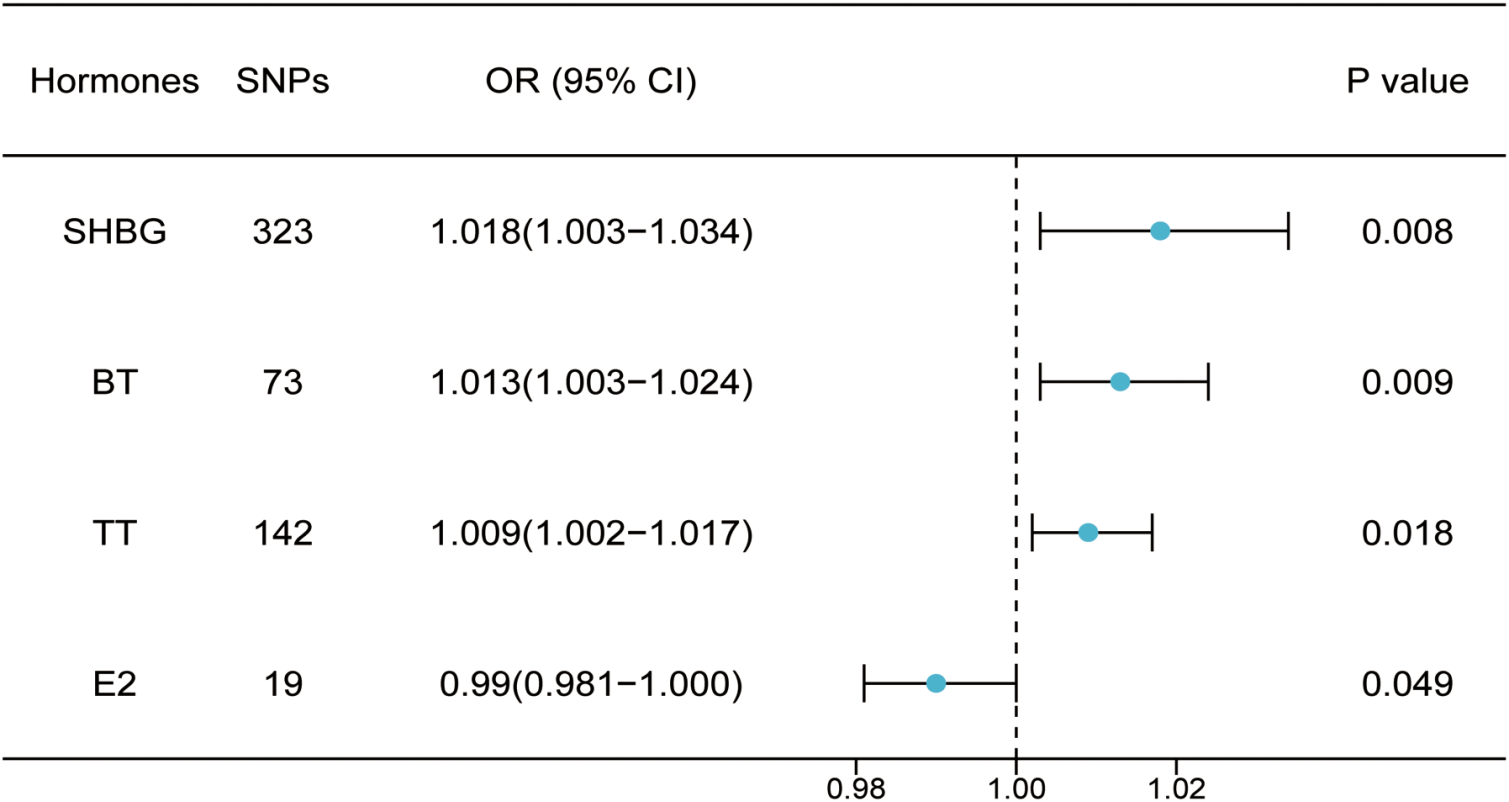
Two-sample MR analysis result of related hormone.

**Figure 4:**
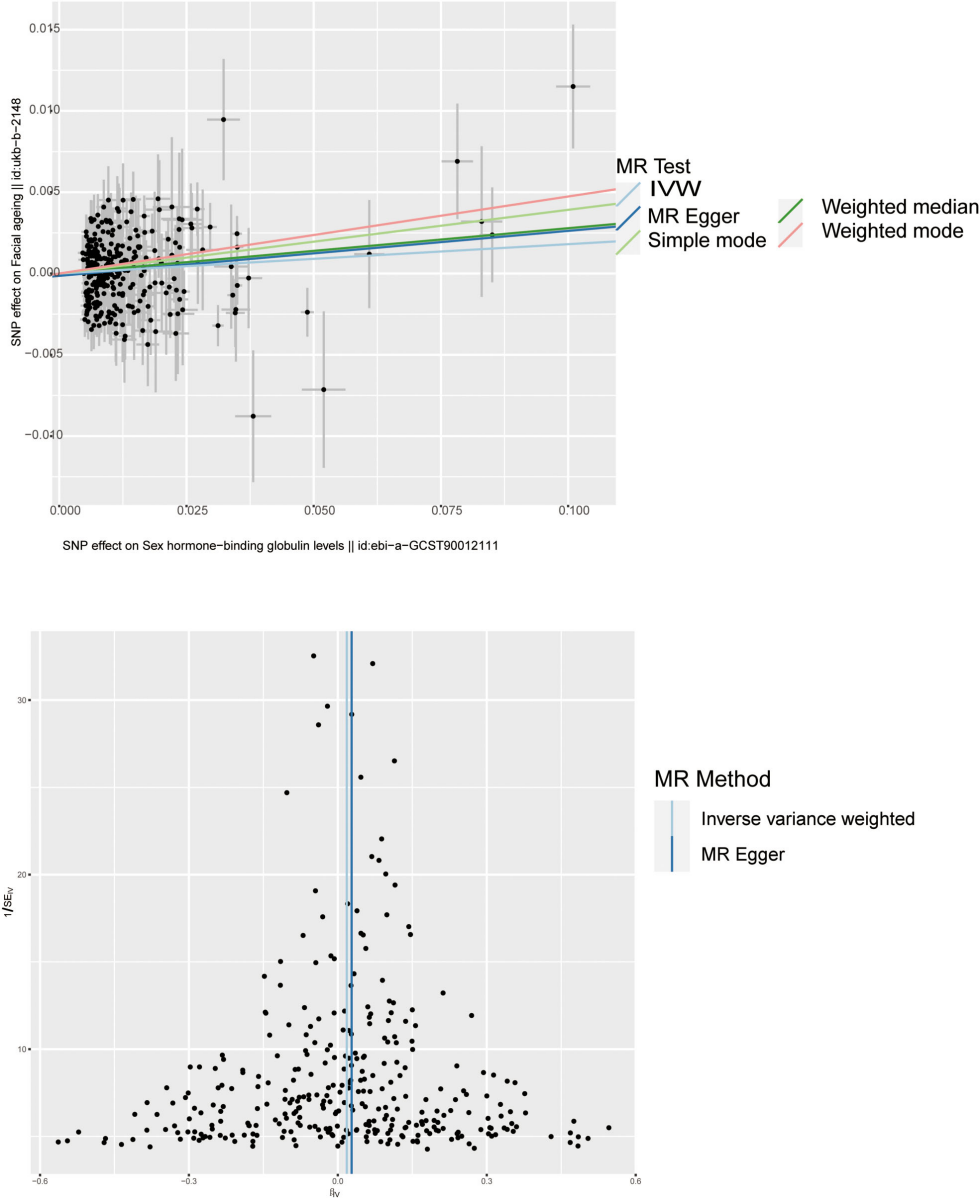
Scatter plot and funnel plot of genetic causality between SHBG and facial aging. The color of the line represents the causality of the different methods.

Through threshold-based filtering, we identified 323 SNPs in *SHBG*. Using the IVW approach, we found that with increasing SHBG levels, the risk of facial aging increased (*p* = 0.035, OR: 1.017, 95% CI: 1.001–1.032). The other methods corroborated that SNPs are positively correlated with facial aging (MR Egger *p* = 0.044, OR: 1.029, 95% CI: 1.001–1.058; weighted median *p* = 0.010, OR: 1.028, 95% CI: 1.007–1.050; weighted mode *p* = 0.010, OR: 1.049, 95% CI: 1.012–1.087) (**Table 1**; **Supplementary Table 1**; **Figures 3**, **4**; **Supplementary Figures 1**, **2**). Pleiotropy, heterogeneity, and sensitivity tests were used for quality control. The results showed that a high level of heterogeneity existed, whereas pleiotropy was absent. Leave-one-out sensitivity analysis showed that all points were on the same side of zero, indicating that individual SNPs did not affect model selection.

**Table 1:** The result and sensitivity analysis of SHBG.

Method	nSNPs	P	OR	Beta	SE	Heterogenicity Test	Pleiotropy Test
MR Egger	323	0.048	1.028	0.028	0.014	4.019e-10^15^	0.409
Weighted median	323	0.009	1.028	0.028	0.011		
IVW	323	0.019	1.018	0.018	0.008		
Simple mode	323	0.184	1.04	0.039	0.029		
Weighted mode	323	0.014	1.049	0.047	0.019		

### 3.2 Two-sample MR analysis of TT, BT, and E2 and facial aging risk

Through threshold filtering, we identified 142, 73, and 19 SNPs in *TT*, *BT*, and *E2*, respectively. The accumulation effect model of IVW was applied for model analysis. The IVW results were TT (*p* = 0.018, OR: 1.009, 95% CI: 1.002–1.0172), BT (*p* = 0.002, OR: 1.012, 95% CI: 1.002–1.023), and E2 (*p* = 0.049, OR: 0.990, 95% CI: 0.981–1.000), indicating that TT and BT are risk factors for facial aging, whereas E2 is a protective factor for facial aging (**Table 2**; **Supplementary Table 2**; **Figure 3**, **Supplementary Figures 3**-**8**). Pleiotropy, heterogeneity, and sensitivity tests were used for quality control.

**Table 2:** The result and sensitivity analysis of BT, TT, and E2.

	Method	nSNPs	Beta	SE	P	OR	Heterogenicity Test	Pleiotropy Test
BT	IVW	73	0.013	0.005	0.009	1.013	0.003	0.573
TT	IVW	142	0.009	0.004	0.018	1.009	1.28E-106	0.64
E2	IVW	19	-0.01	0.005	0.049	0.99	0.058	0.595

### 3.3 Two-sample MR analysis of GH and IGF-1 and facial aging risk

We obtained 13 SNPs in *GH* and 10 SNPs in *IGF1*. The analytical results showed that the levels of GH and IGF-1 have no effect on facial aging (**Table 3**).

**Table 3:** The result and sensitivity analysis of IGF1 and GH.

	Method	nSNPs	Beta	SE	P	OR	Heterogenicity Test	Pleiotropy test
IGF1	IVW	10	1.10E-05	8.05E-06	0.173	1	0.328	0.544
GH	IVW	13	0.003	0.006	0.63	1.003	0.616	0.082

### 3.4 SMR results

Fifteen genes (*AR*, *BECN1*, *ESR2*, *GPER1*, *ESR1*, *PRLR*, *NR3C2*, *NR1I2*, *ESRRG*, *HSD17B2*, *BECN1*, *MT-ATP6*, *GPER1*, *NCOA2*, and *CHRNA4*) were obtained from the drug bank. In the eQTL data, only seven of these genes were found (*BECN1*, *ESR2*, *GPER1*, *ESR1*, *PRLR*, *NR3C2*, and *NR1I2*). The corresponding *p*-values were corrected after SMR analysis, and no relevant genes were related to facial aging (**Table 4**).

**Table 4:** The SMR analysis result of sex hormone-related drug target genes.

Gene	topSNP	Freq	SMR Beta	SMR SE	SMR P	HEIDI P	nSNPs
BECN1	rs1011157	0.109	-0.003	0.004	0.41	0.25	20
ESR2	rs915057	0.4	0.011	0.006	0.068	0.103	20
GPER1	rs10262232	0.217	-0.002	0.005	0.726	0.522	20
ESR1	rs3020333	0.468	-0.015	0.009	0.089	0.134	20
PRLR	rs6451196	0.38	-0.004	0.007	0.541	0.94	20
NR3C2	rs6817545	0.429	0.007	0.014	0.621	0.503	9
NR1I2	rs3732357	0.268	-0.02	0.023	0.39	NA	NA

NA means None.

## 4 Discussion

Using several MR estimation approaches, we investigated correlations between sex hormone levels and facial aging. The results indicated that SHBG, TT, and BT are risk factors, whereas E2 is a protective factor for facial aging. These results provide further evidence supporting the causal role of the above-mentioned hormones in facial aging. In contrast to previous findings *in vitro* (23), this study revealed no evidence of correlations between GH and IGF-1 levels and facial aging.

Aging is an inescapable process, but it can be slowed down, particularly facial aging (24, 25). Human facial aging is increasingly being studied; however, to our knowledge, no studies have investigated the roles of sex hormones in facial aging using GWAS data. Sex hormone levels are thought to fluctuate with age and to be associated with facial aging, and testosterone and bioavailable testosterone levels decline with age, particularly in men (26). Estradiol levels also decline with age, with a rapid decline in women after menopause (27, 28). Therefore, these hormones are considered to be important factors in aging (29). Our results are consistent with this idea and provide new evidence for the influence of these hormones on facial aging.

GH and IGF-1 have been associated with skin aging in previous studies (30), but results were controversial. In the skin, IGF1 promotes hair follicle growth, provides photoprotection of the end hairs, and increases the cell renewal rate of the hair follicles that produce them, thus delaying aging (31). However, Brown et al. (32) found that IGF-1 promoted the development of perioral wrinkles by inhibiting the ability of fibroblasts to eliminate reactive oxygen species. Low GH levels are thought to be related to aging, and GH-treated cells from old mice showed decreased reactive oxygen species production. Macrophage adhesion to laminin and fibronectin substrates was increased in aged mice (33). In addition, cells obtained from older mice showed higher migration rates than those of younger mice, macrophage migration was significantly increased under GH stimulation, and skin fibroblasts isolated from GH mutants were more resistant to various cytotoxic drugs, glucose deprivation, and oxidative damage inducers (34). We used MR analysis, which minimizes the influence of interfering factors on the results, such as photoaging, which is difficult to avoid in experiments, to ensure reliable results. Our results indicated that IGF-1 and GH may not be significantly associated with facial aging. We speculate that (1) this result may also be caused by confounding factors, and (2) this may be because the regulation of GH and IGF-1 on the human body is a complex process, where different effects are expressed in different pathways, which may manifest as promotion in pathways such as hair follicles, and inhibition in certain other pathways. In addition, we performed SMR analysis of action-target genes of hormone-related drugs; however, the results showed that these genes did not significantly affect facial aging.

To our knowledge, this is the first study to use MR analysis of GWAS data to study the effects of multiple hormones on facial aging. We found that SHBG, TT, BT, and E2 can affect facial aging, whereas GH and IGF-1 do not, which is different from findings in previous studies. On the basis of MR analysis, we innovatively used SMR analysis to assess whether hormone-related drug target genes can affect facial aging.

This study had some limitations. Despite the strict screening of SNPs, heterogeneity still existed; therefore, we selected a random-effects model to reduce the occurrence of errors. The results were not satisfactory when using the complementary method, but considering that the IVW method is the most apt and recognized method, we believe that our results are reliable. In addition, facial aging data were collected in the form of questionnaires rather than objectively assessed based on skin swelling or facial wrinkles; therefore, bias cannot be excluded or stratification is not possible. In this regard, our findings need to be validated in studies using objective assessments in other populations (35, 36).

## 5 Conclusion

Our study provided new evidence to support the causal roles of SHBG, TT, BT, and E2 in the development of facial aging, providing a new direction for delaying facial aging. It also revealed that GH and IGF-1 have no causal relationship with facial aging, which is different from previous study findings. This may be explained by insufficient sample size and excessive confounding factors. The mechanisms of these hormones in the process of facial aging require further exploration.

## Data availability statement

The original contributions presented in the study are included in the article/**Supplementary Material**. Further inquiries can be directed to the corresponding authors.

## Author contributions

ZMJ: Writing – review & editing, Conceptualization, Methodology, original draft, Project administration; LHY: Writing – review & editing, Conceptualization, Methodology; ZYS: Writing – review & editing, Formal Analysis; contributed to the revising and review of the article. ZHL: Writing – review & editing; QHZ: Writing – review & editing. All authors contributed to the article and approved the submitted version.

## Supplementary material

Supplementary Figure 1 Forest map of facial aging risk based on SHBG genetic variants. The black line represents the estimated 95% confidence interval.

Supplementary Figure 2 Forest plot for leave-one-out sensitivity analysis of SHBG. The point is completely to the left of 0, indicating that the estimated result from this SNP is that SHBG can reduce facial aging. All points are on the side of 0, representing the stability of the results.

Supplementary Figure 3 Forest map of facial aging risk based on TT genetic variants.

Supplementary Figure 4 Forest plot for leave-one-out sensitivity analysis of TT.

Supplementary Figure 5 Forest map of facial aging risk based on BT genetic variants.

Supplementary Figure 6 Forest plot for leave-one-out sensitivity analysis of BT.

Supplementary Figure 7 Forest plot for leave-one-out sensitivity analysis of E2.

Supplementary Figure 8 Forest map of facial aging risk based on E2 genetic variants.
