# Supplementary figures and images for: Associations between genetically predicted sex and growth hormones and facial aging in the UK Biobank: a two−sample Mendelian randomization study
Source: Front Endocrinol (Lausanne). 2023 Oct 17;14:1239502. doi: 10.3389/fendo.2023.1239502 (PMC10616234; doi:10.3389/fendo.2023.1239502)

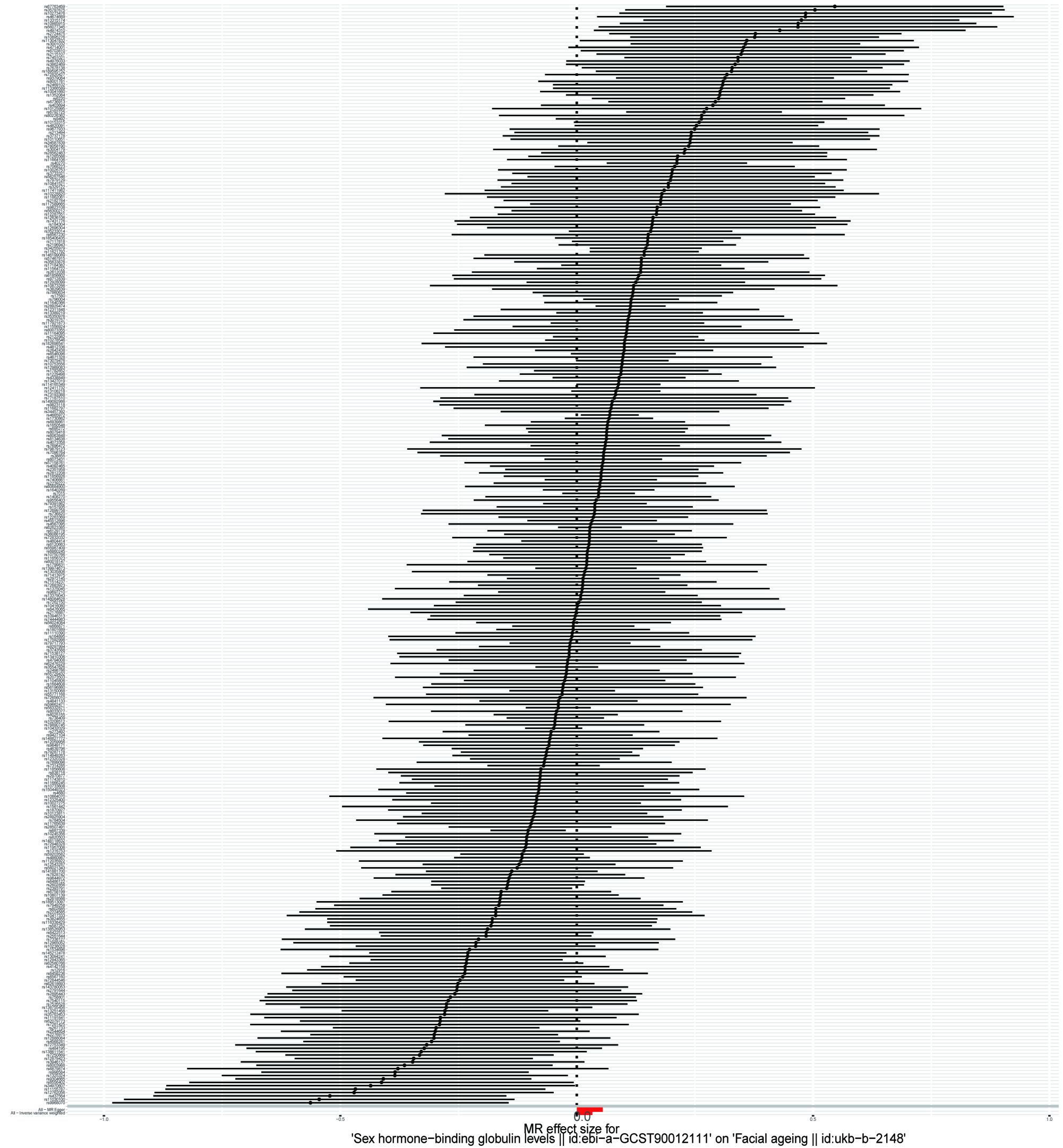

Supplement: Supplementary Figure 1 — Forest map of facial aging risk based on SHBG genetic variants. The black line represents the estimated 95% confidence interval. [file Image_1.tif]

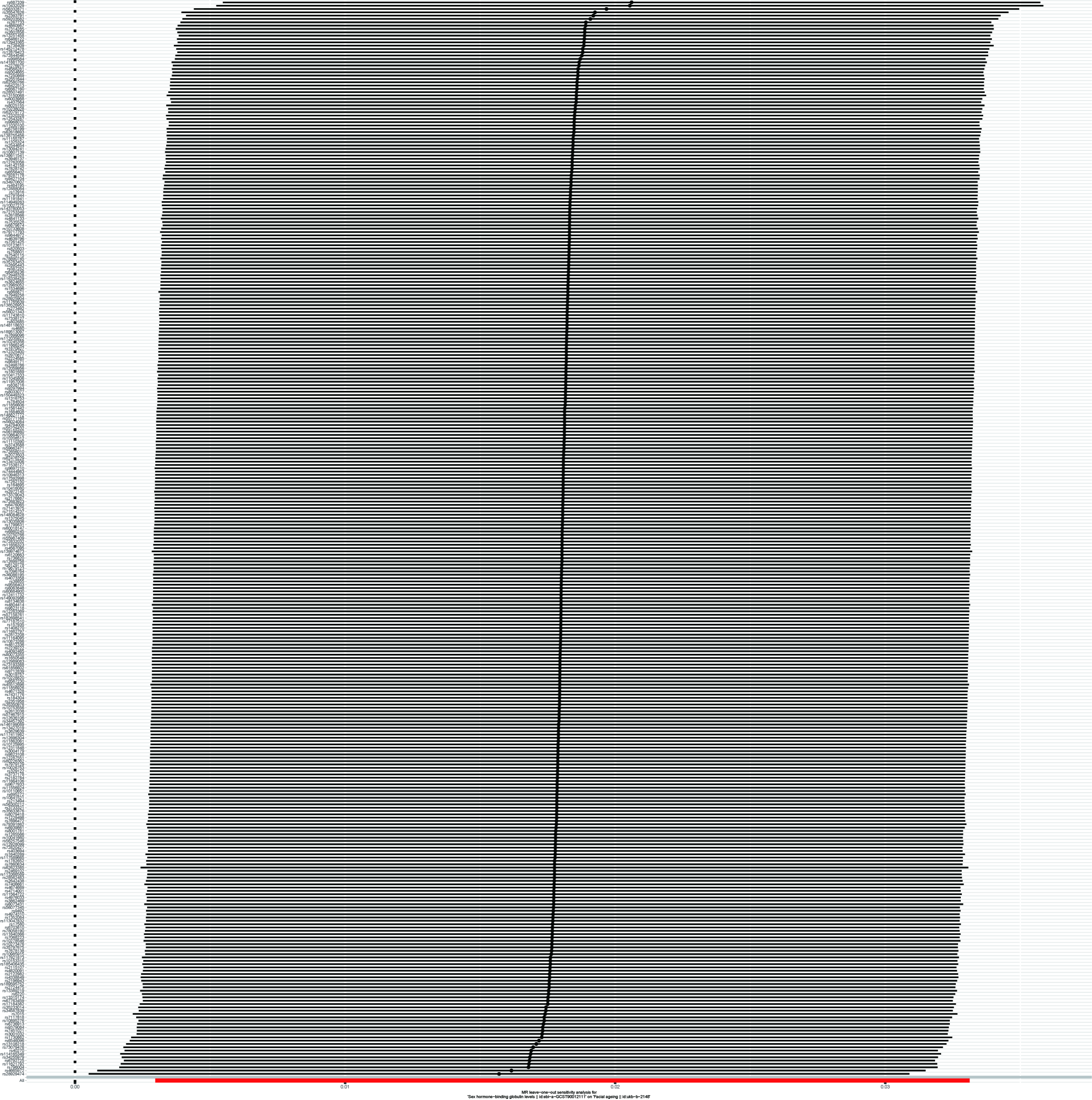

Supplement: Supplementary Figure 2 — Forest plot for leave-one-out sensitivity analysis of SHBG. The point is completely to the left of 0, indicating that the estimated result from this SNP is that SHBG can reduce facial aging. All points are on the side of 0, representing the stability of the results. [file Image_2.tif]

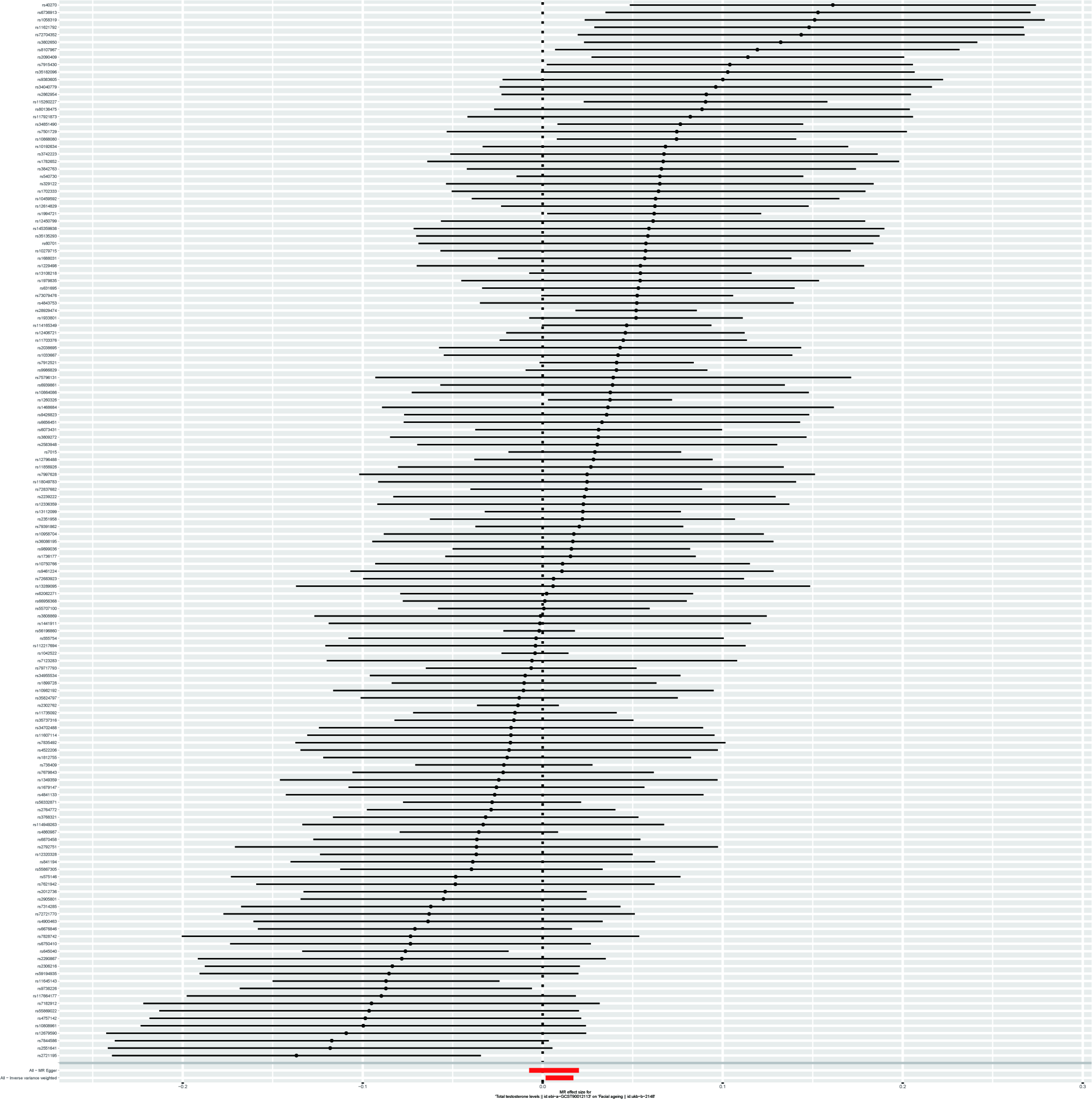

Supplement: Supplementary Figure 3 — Forest map of facial aging risk based on TT genetic variants. [file Image_3.tif]

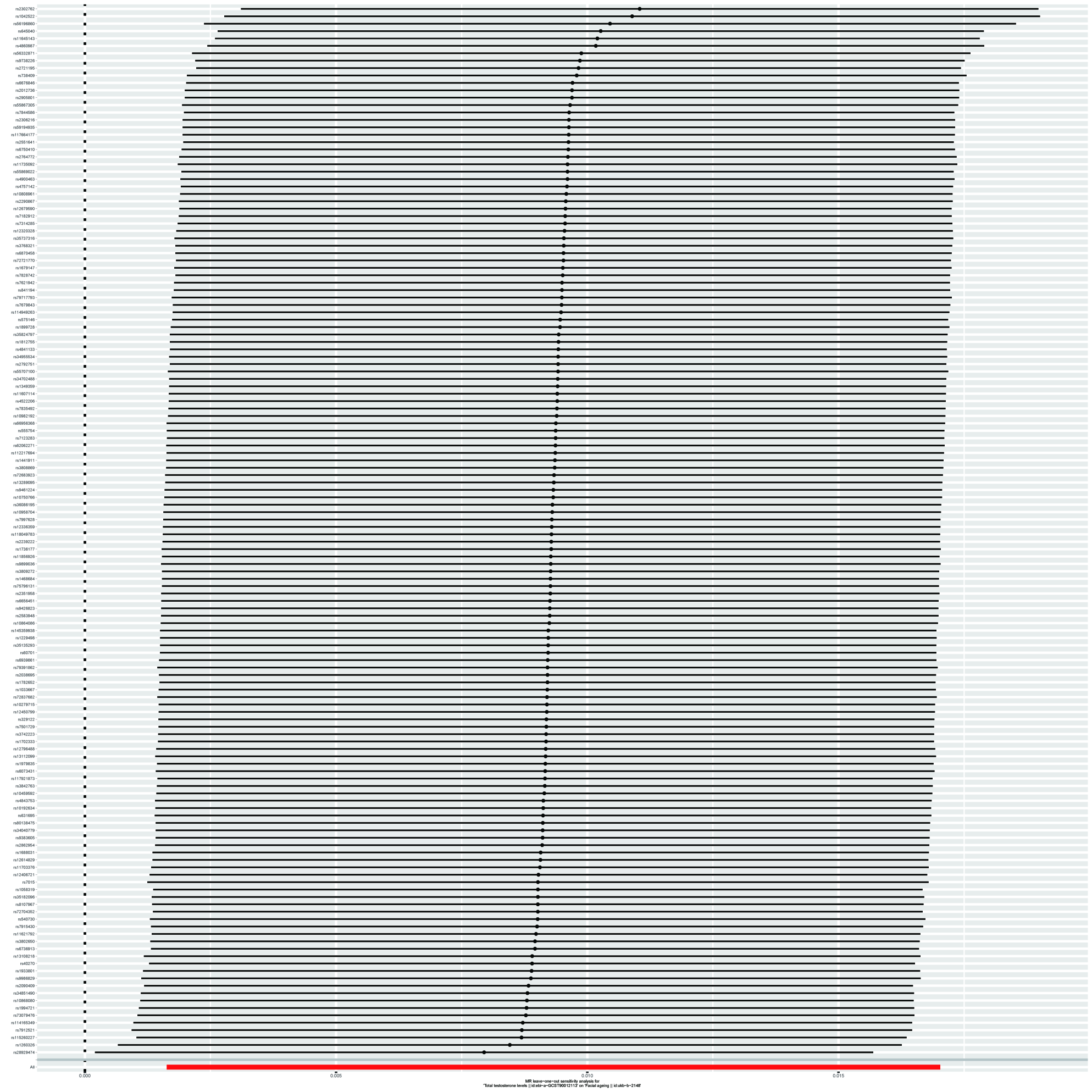

Supplement: Supplementary Figure 4 — Forest plot for leave-one-out sensitivity analysis of TT. [file Image_4.tif]

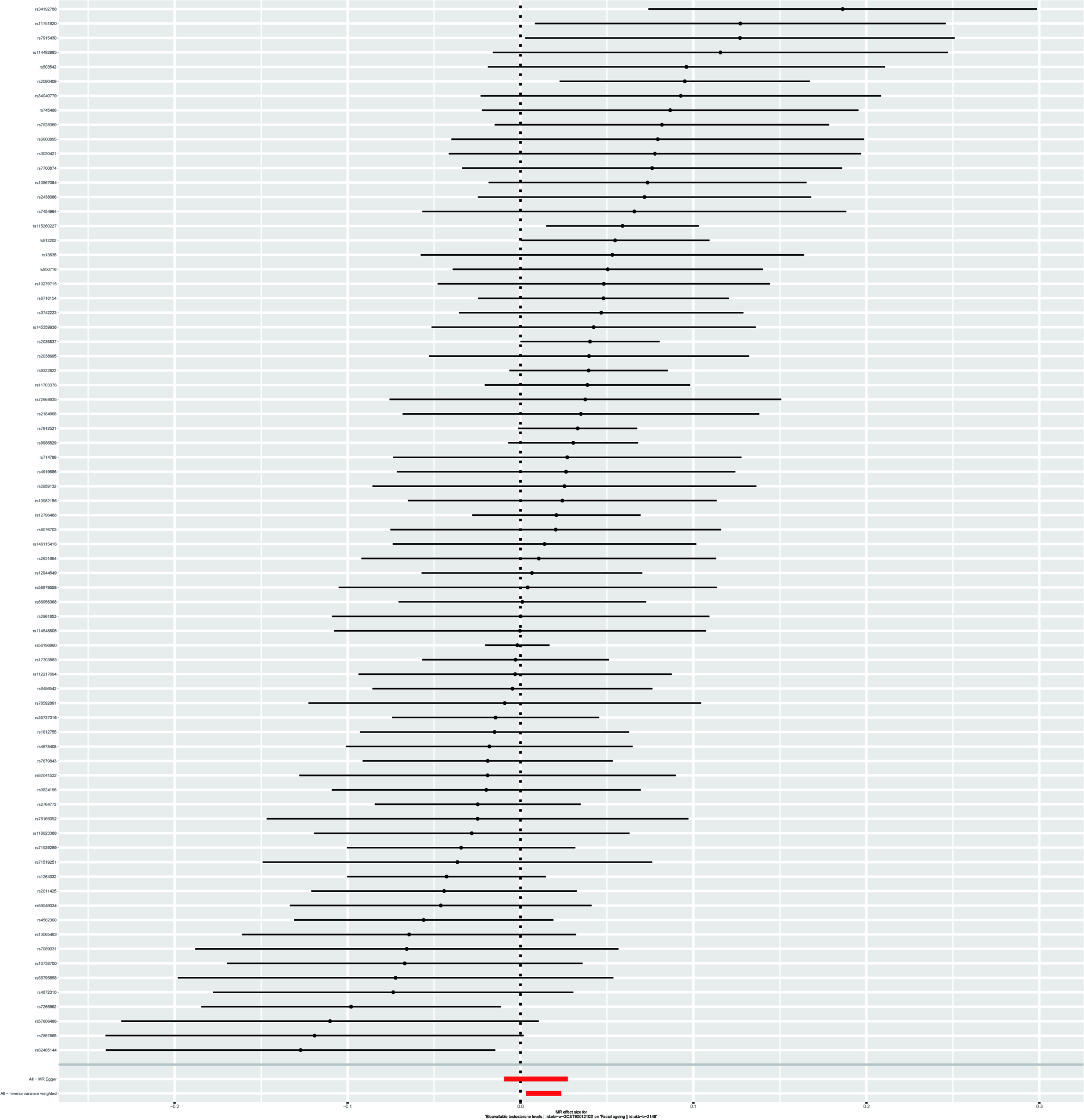

Supplement: Supplementary Figure 5 — Forest map of facial aging risk based on BT genetic variants. [file Image_5.tif]

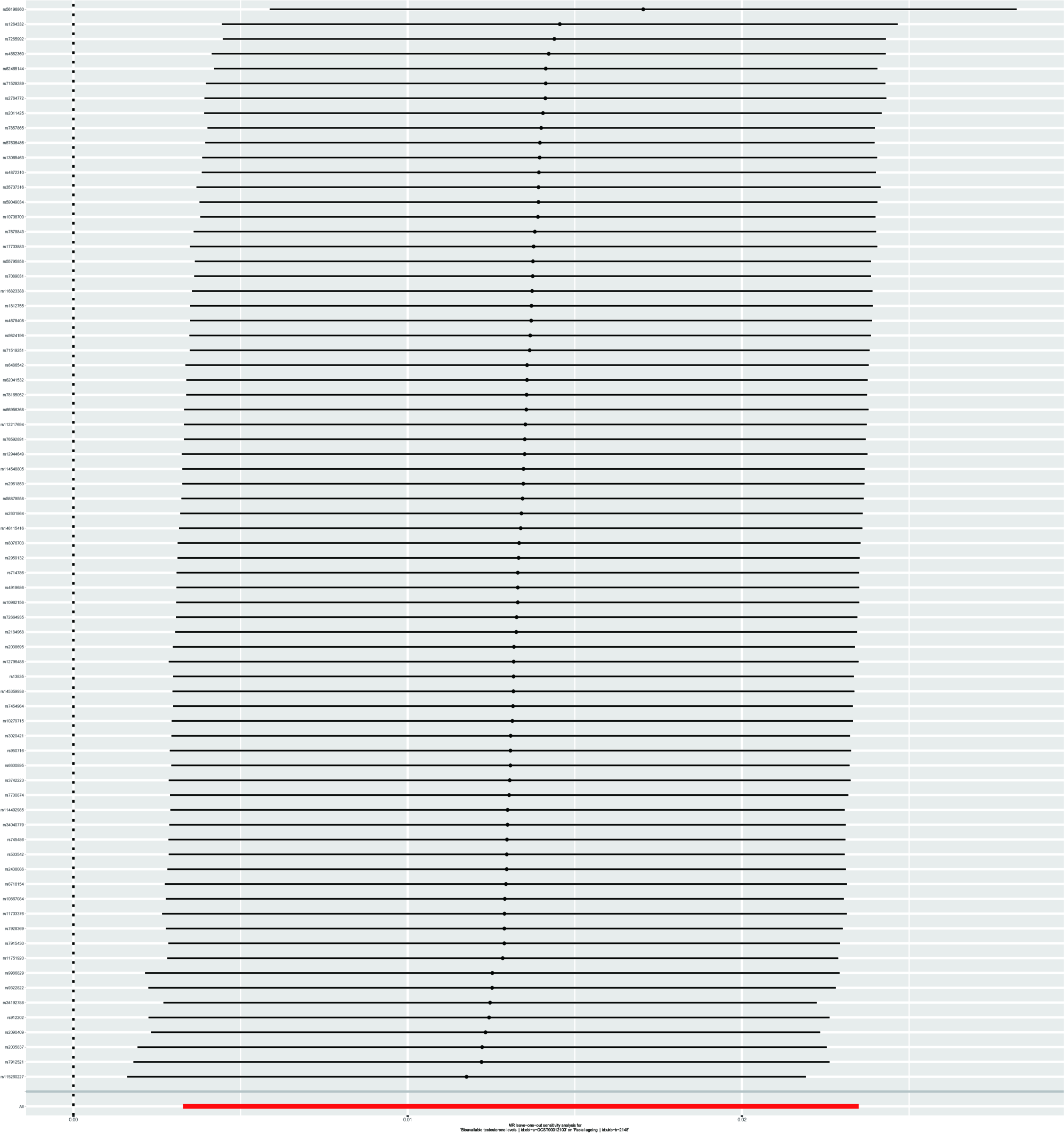

Supplement: Supplementary Figure 6 — Forest plot for leave-one-out sensitivity analysis of BT. [file Image_6.tif]

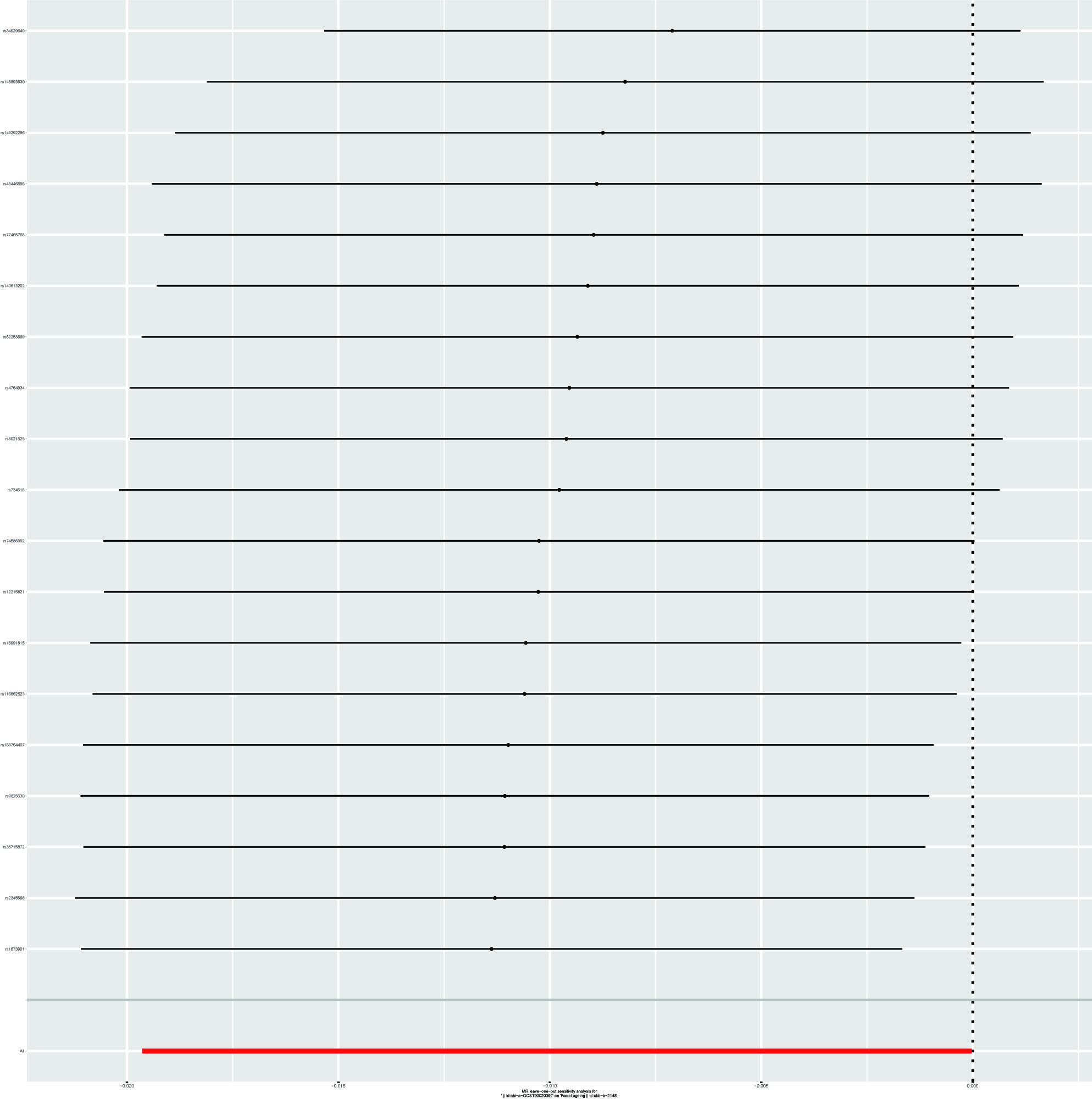

Supplement: Supplementary Figure 7 — Forest plot for leave-one-out sensitivity analysis of E2. [file Image_7.tif]

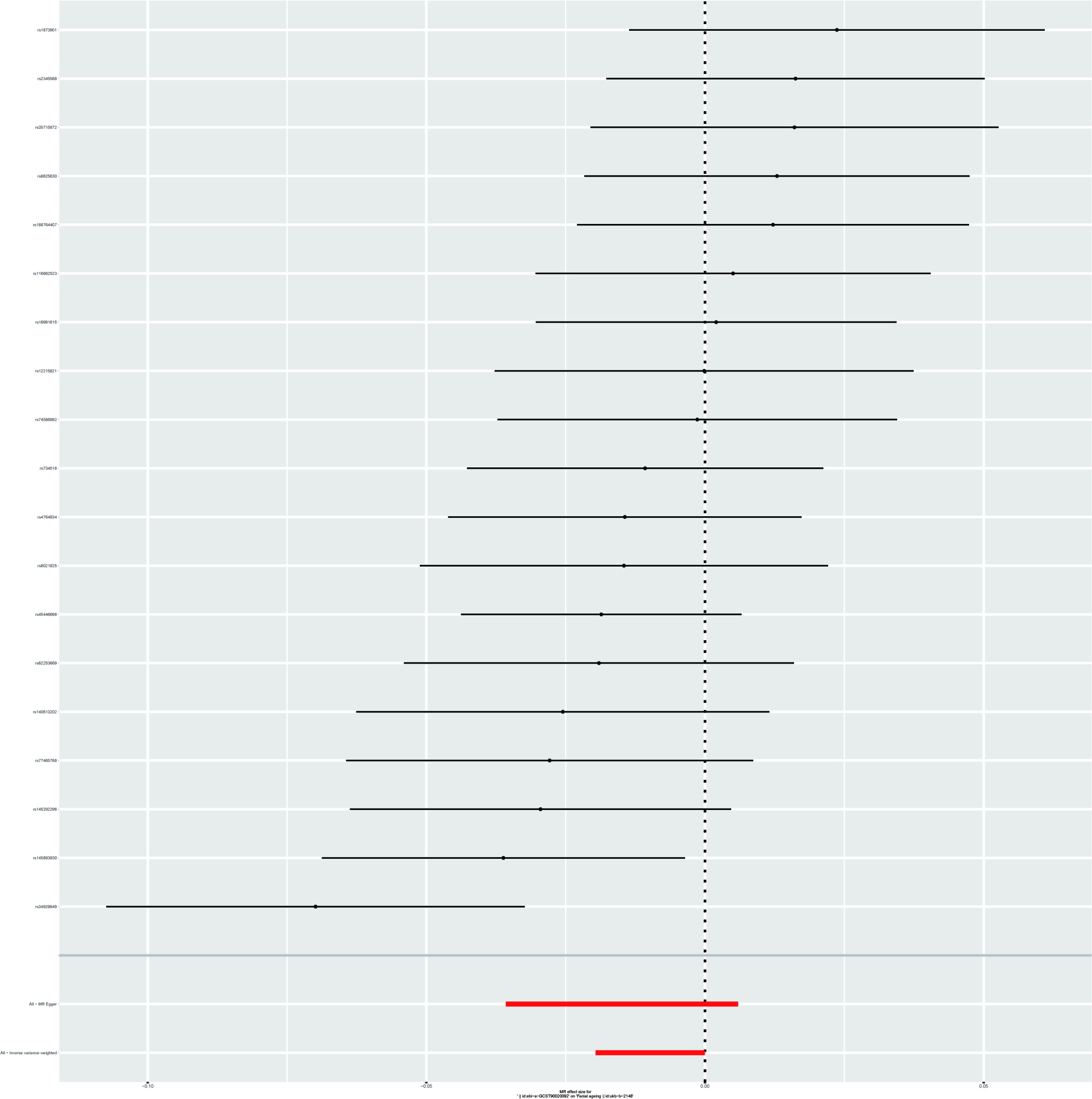

Supplement: Supplementary Figure 8 — Forest map of facial aging risk based on E2 genetic variants. [file Image_8.tif]
